# A perspective: Regeneration of soft and hard tissues in the oral cavity, from research to clinical practice

**DOI:** 10.3389/fdmed.2023.1242547

**Published:** 2023-08-16

**Authors:** Israel Puterman, Matthew J. Fien, Juan Mesquida, Ignacio Ginebreda, Guillermo Bauza, Martha Somerman

**Affiliations:** ^1^ International Dentistry Research Group, Palma de Mallorca, Spain; ^2^ Private Practitioner, Chevy Chase, MD, United States; ^3^ Private Practitioner, Fort Lauderdale, FL, United States; ^4^ Private Practitioner, Palma de Mallorca, Spain; ^5^ Private Practitioner, Barcelona, Spain; ^6^ Center for NanoHealth, Institute of Life Sciences, Swansea University Medical School, Swansea, Wales, United Kingdom; ^7^ National Institute of Dental Craniofacial Research, National Institutes of Health, Bethesda, MD, United States

**Keywords:** dental implants, growth factors, periodontal disease, oral soft tissues, scaffolds, periodontal regeneration, guided tissue regeneration, tissue engineering

## Abstract

Regenerative medicine has gained much attention and has been a hot topic in all medical fields since its inception, and dentistry is no exception. However, innovations and developments in basic research are sometimes disconnected from daily clinical practice. This existing gap between basic research and clinical practice can only be addressed with improved communication between clinicians, academicians, industry, and researchers to facilitate the advance of evidence-based therapies and procedures and to direct research to areas of clinical need. In this perspective, six participants with strong clinical and research interests debated five previously conceived questions. These questions covered current methods and procedures for soft and hard tissue regeneration in the oral cavity with predictable outcomes, limitations of their respective protocols, and needs for future development of regenerative materials and technologies.

## Introduction

This perspective is based on an innovative, interactive video meeting where six researchers and clinicians convened to discuss current strategies and procedures being used for soft and hard tissue regeneration in the oral cavity. Five questions were sent out prior to the session, focusing on addressing which methods and materials provide predictable treatment and identifying the limitations of each protocol. Also discussed were future tools, technologies, and materials required to advance the care of the communities we serve.

A brief background on the six participants in this perspective is provided in Table 1. All of the individuals listed, except for MS, belong to the International Dentistry Research Group (InDent Research), a private initiative founded in 2020 by JM and GB, who recognized a critical need for bidirectional communication between clinicians, academicians, and researchers to facilitate the advancement of evidence-based therapies and procedures and to inform researchers of areas where more evidence-based data are needed to support decision-making at the clinical level. It is noteworthy that GB, the only non-clinical person in the group with a research focus on regenerative medicine, is committed to the goals of InDent Research and visits with regularity the offices of JM and IG to further his understanding of regenerative procedures at the clinical level.

**Table 1:** Zoom debaters.

Name, current positions	Background
Martha Somerman DDS, PhD Field Chief Editor, Frontiers in Dental Medicine	DDS, New York University, USA; Certificate in Periodontology, Eastman Dental: PhD, University of Rochester, NY, USA. Retired May 2021, National Institute of Dental and Craniofacial Research, National Institute of Arthritis and Musculoskeletal and Skin Diseases, National Institutes of Health. Her research focus is on the development and regeneration of tissues of the periodontal complex and bidirectional oral-systemic integration from basic research to health care delivery.
Ignacio Ginebreda, DDS. Private practice, Barcelona, Spain. Medical Director at InDent Research.	School of Dentistry graduate at Universitat Internacional de Catalunya, Barcelona, Spain. Implantology training program at the University of California, Los Angeles, CA, USA. In private practice since 2014, with research focused on implantology and guided tissue regeneration using different materials, such as synthetic bone substitutes.
Israel Puterman, DMD, MSD. Private practice, Chevy Chase, MD, USA. Co-Medical Director at InDent Research.	Boston University graduate in dentistry, dual program in implant dentistry and periodontics at Loma Linda University, CA, USA. In private practice since 2008 with a focus on tissue regeneration, soft and hard tissues, and implants. An active member of the American Academy of Periodontology and the Academy of Osseointegration.
Matthew J Fien, DDS Private practice, Fort Lauderdale, FL, USA. Co-Medical Director at InDent Research.	BSc graduate from the University of Florida and doctorate from Columbia University School of Dental and Oral Surgery, with post-graduate training in periodontology at Nova Southeastern University. In private practice for 15 years, with interest in clinical research studies in the fields of periodontics, implant dentistry, and tissue regeneration. An active member of the American Academy of Periodontology and the Academy of Osseointegration.
Juan Mesquida, DDS Private practice, Chevy Chase, MD, USA. Co-Medical Director and Founder of InDent Research.	Universidad Alfonso X El Sabio (Madrid, Spain), graduated in Dentistry in 2005. Masters in Implant Dentistry in ESI Barcelona, Spain. Specialist in Implant Dentistry, Advanced Education in Implant Dentistry, Loma Linda, CA, USA. Former assistant professor at the same program. International lecturer. In private practice since 2014 in Palma de Mallorca, Spain. Practice focused on surgical and restorative aspects of implant dentistry. Co-founder of Indent Research.
Guillermo Bauza, PhD Hon. Lecturer at the Faculty of Medicine, Health and Life Science at Swansea University, Swansea, UK Research Director and Co-founder of InDent Research, Mallorca, Spain.	BSc in Medical Genetics at Swansea University, Wales, UK. PhD joint program in tissue engineering and regenerative medicine at the Houston Methodist Research Institute, TX, USA, and Swansea University, focused on musculoskeletal regeneration using stem cells and biomimetic scaffolds. InDent Research's co-founder and research director since 2020.
International Dentistry Research Group (InDent Research) www.indentresearch.com info@indentresearch.com @indent_research	A private initiative launched in 2020 to serve as an international hub and conduct high-quality research to transform clinical practice and bridge the existing gap with research. Formed by clinical and fundamental investigators to conduct innovative research and to bring robust scientific evidence to the treatments and outcomes for patients, fostering collaborations as a key element to success.

## Debate questions/topics

### What are the current regenerative therapies you are using in your practice? If applicable, please include approaches you use based on age, gender, compliance, health status, and medications.

The practices of all the clinicians on the video call focus on the regeneration of both soft and hard tissues, with some practices having a large clinical need for implant-related tissue regeneration. In general, there was agreement on the types of techniques and materials used and that the specifics of a given procedure are based on the individual patient, taking into consideration health status, compliance, age, medications, type of defect, and product availability in the geographic area, in addition to country regulations. Regenerative procedures are frequently used for ridge augmentation, immediate implant placement, socket/ridge preservation, and regeneration of tissue lost to disease, trauma, and infection. Therapies selected by the clinicians surveyed are primarily based on the type of defect, with routine use of growth factors and biologics in complex cases, such as immunocompromised patients and patients with complex and large defects, with the aim of providing an improved healing environment and promoting a regenerative cellular response.

Consistently expressed by the clinicians was the need for materials and membranes that ensure predictable primary wound closure. Of the currently available materials (1–3), the preference is for resorbable materials. However, they do support the use of non-resorbable membranes for vertical augmentation procedures. The limiting factor for using biologics was mostly the cost. Guided bone regeneration (GBR) appears to be the standard of practice, and it includes the use of allografts, xenografts, or synthetic bone grafts combined with autograft bone plus a membrane (4, 5).

Specific factors are based on the scientific literature and conferences attended, acknowledging that these factors promote the positive regeneration of soft and hard tissues. Factors mentioned included: autologous blood-derived products, such as platelet-rich fibrin (ABPs/PRF) (6); recombinant human platelet-derived growth factor-BB (rhPDGF) (7); enamel matrix derivative (EMD) (8); and bone morphogenetic proteins (rhBMP2/BMPs) (9), although the latter is more commonly used by oral and maxillofacial surgeons for larger defects (10). Further, it was mentioned that BMPs are expensive and thus cost-benefit needs to be considered in the decision-making process (11). Regarding soft tissue grafts, in addition to autografts, some clinicians use collagen-based membranes (12) along with rhPDGF or EMD to promote cell migration and proliferation in addition to vascular infiltration, as reported in the literature (3). More recently, two of the clinicians (MF and IP) have been using amnion-chorion grafts (13), which are known to be rich in biological factors and also reported to be antimicrobial, with some positive results to date (14–18). All clinicians agreed that they would prefer allografts or xenografts to connective tissue grafts if predictability, both esthetically and in terms of gaining attached gingiva, could be achieved.

The use of lasers as a noninvasive procedure to remove bacteria and promote wound healing was briefly discussed, but it is not routinely utilized by InDent Research clinicians. Concerns about the use of lasers included a learning curve due to a lack of tactile sensitivity and a lack of predictability of positive outcomes (19, 20). Patient compliance was cited as a possible explanation for the lack of positive outcomes.

Patients' medications must be considered as they may affect the wound healing process in general and specifically the regeneration of soft and hard tissues (21). Some examples include osteoclast regulatory factors, which are used to treat osteoporosis and other mineralized tissue disorders (22), chronic inflammatory conditions (23), cancer patients (24), those receiving monoclonal antibody therapy (25), or selective serotonin reuptake inhibitors (26). Clinicians agreed that the use of such medications does not impede indicated surgical procedures but may alter the schedule and recovery times from a procedure, in addition to the selection of a specific therapy. There was also mention of the need to monitor blood levels of vitamin D, which is involved in bone health modulation and wound healing in general (27, 28). Moreover, a standard blood profile may be useful to monitor general health status. Uncontrolled diabetics and heavy smokers are not considered good candidates for regenerative therapies, as reinforced by the literature on this subject (29, 30).

Regarding age, there was agreement that the predicted life span of a material and a patient, plus the patient's tolerance to longer procedures, needed to be considered. For example, a less expensive procedure or therapy with a limited lifespan may be indicated for older patients with compromised health, but longer-lasting materials may be preferred for younger and healthier patients.

Listening to the clinicians discuss current technologies, it was apparent that, like researchers, they use evidence-based data points. However, clinicians' data points consider the patient in the chair and include compliance (a significant factor mentioned by all), the complexity of the defect, biomechanical properties, medical and dental history, and age (31, 32). These concerns need to be taken into consideration when developing materials, membranes, or medical devices. The InDent Research group felt that there was a need for an improved dialogue between the researcher and their biological approach to designing materials, devices, and technologies, and the clinician's approach to the selection of therapies, which importantly includes the different profiles of the patient in the chair.

### Do you feel that existing regenerative approaches have robust and predictable outcomes?

There was agreement that the current approaches and materials used to promote tissue regeneration have improved considerably over the past decade and, as mentioned above, are routinely used with a case-by-case approach and are situation-specific in terms of predictability (33). All InDent Research clinicians use regenerative procedures weekly, most of them daily, and are satisfied with the clinical outcomes. Predictability centered on patient compliance, oral hygiene, health status, and defect types, with less success in vertical defects where non-resorbable membranes are indicated (5), and in situations where primary closure was not maintained. As emphasized throughout the session, for procedures to be successful, primary closure of the surgical site is critical (34), especially in non-compliant patients, and all agreed that with the materials available at this time, resorbable membranes provide a better chance of maintaining wound closure than non-resorbable materials. There was consensus that improved non-resorbable membranes with increased osteoconductivity, along with resorbable membranes with improved biologics, are needed to achieve and maintain primary closure.

At the time of addressing this question, an issue arose regarding the incompleteness of many systematic reviews and their limited inclusion of publications due to a high risk of bias, providing minimal statistical significance, and thus not being valuable for clinicians (35). There was agreement that many existing systematic reviews are of limited value because of insufficient data points, e.g., the lack of shared electronic records and thus a lack of means to track procedures, and limited randomized clinical trials, in part related to the high cost of such trials and thus limited funding in this area. Also mentioned was the need for stringent guidelines for determining the acceptance or rejection of systematic reviews and for reviewers of such articles to include at least one clinician in full-time practice. Table 2 and the Conclusion section include suggested approaches to advance the exchange of ideas between researchers and clinicians.

**Table 2:** Suggested mechanisms for advancing communications between the research and clinical communities.

Manuscript reviews	For submissions having a clinical focus, at least one clinician and one researcher should be appointed as reviewers. Similarly, research articles should always include a dental practitioner in their authorship providing a clinical implication perspective of the study.
Study groups	Many dental study groups exist with the active participation of clinicians in case of revision, offering advice based on experience. Adding researchers to the group sessions would add scientific rigor to the statements, and information to the clinicians on emerging areas of research, and identify clinical areas where further research is needed.
Meetings	At both research and clinical meetings, such as the International Association of Dental Research, European Periodontology meeting, Gordon conferences, European Association for Osseointegration, and American Association of Dental, Oral, and Craniofacial Research, at least one session or symposium per meeting should focus on dialogue between clinicians and researchers on specific topics, and such symposia should be published or recorded.
Journals	Several journals have made attempts to bring the clinical and research communities together such as the MDPI, with a special issue on Clinical Applications and Fundamental Research in Dentistry; the journal Clinical Oral Investigations, the International Association for Dental Research (IADR) with the JDR Clinical and translational research; the American Dental Association (ADA), with their newer journal JADA Foundational Science; newer journals such as Frontiers in Dental Medicine, Frontiers in Oral Health and many more. More efforts in this direction are required.
Patient engagement	A systematic patient record including patient pre- and post-surgery surveys with treatment expectations and post-surgical impressions could increase the clinician's understanding of patients’ needs and treatment limitations.
Continuing education (CE)	Clinician affiliation with a research institution and rotation through a research department at least one or two months every year and reporting research engagement during the period. Also, researchers should engage in at least one clinical opportunity per year, such as clinical teaching; dental study clubs; clinical meetings; dental CE, either in person or in a videoconference.

### In your practice, how often do you currently use these therapies and/or plan to use them in the future: factors (biologics and/or synthetics); cell therapies; cell-free therapies; scaffolds; and gene therapy. Also, looking to the future, which of these therapies and/or others do you think hold promise for more predictable and optimal results?

As stated above, biologics and scaffolds are routinely used in clinical practice. Biologics used that were mentioned during our video call included EMD, rhPDGF, ABPs/PRF, and rarely BMPs due to cost/benefit and lack of availability in certain regions of the world. In terms of scaffolds or bone substitutes, allografts, xenografts, and synthetic bone substitutes are commonly used in surgery with predictable results. Collagen xenograft or allograft and amnion-chorion membranes are also utilized with promising results; however, the latter membranes are not available in many regions of the world (12–16). It is important to note that the regulatory agencies in Europe are restrictive on the use of biologics and allografts, and differences between EU countries exist, making it difficult for clinicians to use many of these materials across Europe (36).

A recurrent theme was the routine use of biologics for complex cases (e.g., defect complexity, patient compliance, medical and dental history) and was supported by the literature indicating that biologics promote an optimal environment for wound healing, although it was recognized that some of the current data are conflicting and, further, that existing materials are not as robust nor predictable as desired for their patients (37–40). In short, the American Academy of Periodontology consensus statement, based on existing data and expert opinion, was that the biologics they considered (EMD, rhPDGF, ABPS, and rhBMP2) were safe and provided added benefits over conventional treatments (40). However, as mentioned by the InDent Research members during the debate session, the benefits and risks are variable and depend on the specific biologics used and patient-related issues, such as the type of defect, patient compliance, and medical/dental history. The InDent Research group agreed that there is no downside to the use of biologics other than cost, and this is discussed with their patients. One clinician stated, as a positive, that this is “moving the research to the patient in the chair”. All agreed that if they were the patient, they would use a biological factor every time based on supporting data, and of course, in their situation, cost would not be an issue.

Among the biologics used, there was some discussion on their use in specific situations. None of the InDent clinicians use platelet derivatives for hard tissue regeneration, citing a lack of confirmation of results in the literature (41). In contrast, two of the clinicians mentioned that they use platelet derivatives for soft tissue procedures with successful outcomes, feeling that platelet derivatives and perhaps other biologics are more effective for soft tissue healing than for hard tissue. However, a third clinician no longer uses platelet derivatives for any procedures because he does not see any benefits based on clinical outcomes with or without this derivative. In terms of scaffolds, and reinforced by the answer to the question above, there was agreement that scaffold materials have improved over the past decade but are still not ideal, as they lack predictability in terms of achieving primary closure and/or the ability to regenerate bone/PDL/cementum (periodontal complex). In terms of BMPs, it was felt that the current delivery system, collagen, is not optimal since collagen lacks the mechanical properties needed to maintain tissue architecture during regeneration (5, 42). As a solution, clinicians are using titanium mesh to support the graft space (43), although the success of these procedures is technique-dependent (5).

There was consensus that future research on materials needs to include improvements in scaffolds that are mechanically stable, provide a better carrier for factor release (e.g., slow release of factors over time), do not cause wound dehiscence, and are easy to handle. There was interest in further communication about the new types of scaffolds currently in clinical trials, such as a new biocompatible synthetic bone adhesive that is reported to be osteoconductive, bioactive, biodegradable (over the long-term, 6–9 months), and to solidify quickly once placed in the defect, offering rapid mechanical stability (44, 45). All agreed that if these properties hold true, such a scaffold will be transformative and much needed to achieve the goals of predictable wound closure and tissue regeneration of the periodontal complex and for applications in implant placement (46–48).

Regarding the use of gene therapy, while this may not be an approach for all patients, there was agreement that this may be a suitable approach for certain individuals as we continue to gain knowledge about the causes of severe forms (aggressive forms) of periodontal disease and, therefore, situations associated with genetic profiles (49, 50). In fact, such an approach is being considered for the treatment of individuals with hypophosphatasia (HPP), a hereditary disease caused by a deficiency of alkaline phosphatase (TNAP). These individuals have defects in bones and teeth, such as root formation or cementogenesis, because increased pyrophosphate inhibits the mineralization of these tissues (51). The extent of the disease varies from mild defects limited to the teeth (odontoHPP) to perinatal HPP disease. Current therapies include the use of bone-targeted enzyme replacement therapy (TNAP), with evidence of successful outcomes for skeletal and dental tissues (52), but patients need weekly injections. More recent studies suggest that viral vector delivery of mineral-targeted TNAP may be a viable approach (51–53). Moreover, data using a rodent periodontal defect suggest that local delivery of TNAP promotes periodontal tissue regeneration (54). Also noteworthy is the publication by S Makawa et al. (55), where using both an *in vitro* approach (cell culture) and an *in vivo* rodent (rat) tooth extraction/implant model and BMP gene immobilization technology, the researchers reported an increase in osteoblast differentiation *in vitro* and positive alveolar bone regeneration *in vivo*.

In reference to the use of cell therapies, one clinician mentioned that he was discussing the use of cell therapies with a company but had not yet explored the clinical approach. However, the Ph.D. member of InDent voiced concern over the use of cell therapies, especially stem cell (SC) therapy, to treat the complex environment within the oral cavity. Although SCs are known to have strong regenerative and anti-inflammatory potential (56, 57), the complex local inflammatory environment within the oral cavity together with the large microbial populations may promote an inconsistent SC response (56, 58), and further research is required on how to condition for an anti-inflammatory SC response (59, 60).

In general, there was interest in and anticipation of next-generation scaffolds, as discussed above, and for a more predictable application of biologics, recognizing that a single factor may not be the solution (61). Rather, as improvements in carriers for factors are achieved, the slow release of two or three factors may be needed (62), such as a first factor that promotes migration to the site and proliferation of cells within the local site, followed by a factor that promotes cell differentiation into an osteoblast-like and cementoblast-like cell.

### Are there any specific surgical techniques, tools, or devices that you use now, and why? Also, are there any specific surgical techniques, tools, or devices you are considering in the future, and why?

In terms of tools and technologies, there was support for new technologies and improvements in existing technologies such as 3D printing, digital technologies, and cone beam computed tomography (CBCT). With continued advances in tools and technologies, the group is confident that there will be less human error in diagnosis, treatment approaches (more predictable), and clinical outcomes. Areas mentioned during the discussion included:

(1) 3D printing: Enabling individualized, custom-fit materials that optimize the delivery of a material to a specific site (63–66).
(2) Scaffolds: New developments, including laminar cortical plates that provide better structure to ensure the needed mechanical proprieties, flexibility, delivery/carrier of factors, and primary closure of defects (11, 61).
(3) Navigated surgery: For the clinicians in the session using this technique, they feel that it has revolutionized their placement of implants, bone harvesting, and more, where there is less stress as they are better informed of nerves and other structures during their surgical procedures. They mentioned that a learning curve is required to be comfortable with the use of the system (67–69).
(4) Artificial intelligence (AI)/Machine learning: There are some existing software systems and more in development that will help inform prevention, diagnosis, and treatment approaches, thus improving the quality-of-care delivery. In a similar fashion to the transition from radiology files and lost images to radiographic images, the group is optimistic that AI and machine learning will transform our practices and allow clinicians to devote more time to direct patient care (70).
(5) Robotics: Although still in the very early stages, some value has been reported for the use of robotics (71, 72).
(6) Other devices—now/future: Also mentioned were the use of lasers and piezoelectric surgical technologies, with some using these now and feeling that, as these technologies improve and others are currently being developed or planned, positive outcomes will be more predictable and there will be an increase in the use of newer technologies (73, 74).

### What areas of research do you think are needed to advance the success of regenerative therapies? This includes tools and technologies. Also, do you have any suggestions on how to increase the interaction between the research/academic community and the practice community to improve clinical outcomes?

This question was largely addressed in the previous sections. In addition, there was some discussion on the potential value of pharmacogenomics and pharmacogenetics (75, 76) using biomarkers, genetic panels, and proteomic profiles derived from biological fluids, e.g., blood, saliva, and crevicular fluid, to personalize dental treatments. For example, the use of biomarkers coupled with machine learning and other emerging tools and technologies can assist in determining which patients will respond best to a given treatment. More recently, epigenetic studies, understood as external factors that influence gene expression (77), have focused on developing biological approaches to redirect degenerative or diseased processes in the oral cavity (78, 79).

However, at this time, the group feels that the available technology may provide some interesting data but will not alter patient care and will be expensive. The current predictors used by clinicians—medical and dental history, plaque and bleeding index, and compliance—offer similar information to that provided by kits available on the market. The group looks forward to further research in this area to provide evidence that changes in the biomarker profile can predict oral-dental health status over time and assist in informing a patient's preventive plan, diagnosis, and response to a given treatment.

## Conclusion

This video call, with clinicians and researchers in attendance, provided an appropriate forum for an open discussion of current regenerative procedures and future directions for advancing predictable regenerative procedures. However, we acknowledge that there were limitations to our debate format. Our virtual discussion was informal and did not require specific instructions, time limits, or polling of each person at the end of a question. In the future, readers who wish to use a debate-type format may want to consider a more formal methodology. All participants remarked on how far we have come in the past decade in improving the availability of tools, devices, materials, and technologies and recognized that these advances would not be possible without research efforts that include academic, clinical, and industry settings.

InDent Research has only been in existence for two years, but there is a consensus that its model, which includes clinicians in both clinical practice and academia, in addition to PhDs with a research focus to complement clinicians in practice, supports dialogue among the various stakeholders. Those in full-time practice stated that most of their information about new products is obtained from industry, corporations, meetings, and selected clinical journals. However, their training and education are focused on the clinical side, so there is a need for better communication with researchers to assist in interpreting data from numerous publications, including systematic reviews.

## Data Availability

The original contributions presented in the study are included in the article/Supplementary Material, further inquiries can be directed to the corresponding author.

## Author's note

MS is a Special Volunteer at the National Institute of Dental Craniofacial Research.
